# Genome-wide identification of genes involved in the positive and negative regulation of acetic acid-induced programmed cell death in *Saccharomyces cerevisiae*

**DOI:** 10.1186/1471-2164-14-838

**Published:** 2013-11-28

**Authors:** Marlene Sousa, Ana Marta Duarte, Tânia R Fernandes, Susana R Chaves, Andreia Pacheco, Cecília Leão, Manuela Côrte-Real, Maria João Sousa

**Affiliations:** Centre of Molecular and Environmental Biology (CBMA), Department of Biology, Universidade do Minho, Campus de Gualtar, 4710-057 Braga, Portugal; Life and Health Sciences Research Institute (ICVS), School of Health Sciences, University of Minho, 4710-057 Braga, Portugal; ICVS/3B’s - PT Government Associate Laboratory, Braga, Guimarães, Portugal

**Keywords:** Phenotypic screen, Euroscarf knock-out mutant collection, Yeast, Apoptosis, Tumour cells, Alcoholic fermentation

## Abstract

**Background:**

Acetic acid is mostly known as a toxic by-product of alcoholic fermentation carried out by *Saccharomyces cerevisiae*, which it frequently impairs. The more recent finding that acetic acid triggers apoptotic programmed cell death (PCD) in yeast sparked an interest to develop strategies to modulate this process, to improve several biotechnological applications, but also for biomedical research. Indeed, acetate can trigger apoptosis in cancer cells, suggesting its exploitation as an anticancer compound. Therefore, we aimed to identify genes involved in the positive and negative regulation of acetic acid-induced PCD by optimizing a functional analysis of a yeast Euroscarf knock-out mutant collection.

**Results:**

The screen consisted of exposing the mutant strains to acetic acid in YPD medium, pH 3.0, in 96-well plates, and subsequently evaluating the presence of culturable cells at different time points. Several functional categories emerged as greatly relevant for modulation of acetic acid-induced PCD (e.g.: mitochondrial function, transcription of glucose-repressed genes, protein synthesis and modifications, and vesicular traffic for protection, or amino acid transport and biosynthesis, oxidative stress response, cell growth and differentiation, protein phosphorylation and histone deacetylation for its execution). Known pro-apoptotic and anti-apoptotic genes were found, validating the approach developed. Metabolism stood out as a main regulator of this process, since impairment of major carbohydrate metabolic pathways conferred resistance to acetic acid-induced PCD. Among these, lipid catabolism arose as one of the most significant new functions identified. The results also showed that many of the cellular and metabolic features that constitute hallmarks of tumour cells (such as higher glycolytic energetic dependence, lower mitochondrial functionality, increased cell division and metabolite synthesis) confer sensitivity to acetic acid-induced PCD, potentially explaining why tumour cells are more susceptible to acetate than untransformed cells and reinforcing the interest in exploiting this acid in cancer therapy. Furthermore, our results clearly establish a connection between cell proliferation and cell death regulation, evidencing a conserved developmental role of programmed cell death in unicellular eukaryotes.

**Conclusions:**

This work advanced the characterization of acetic acid-induced PCD, providing a wealth of new information on putative molecular targets for its control with impact both in biotechnology and biomedicine.

**Electronic supplementary material:**

The online version of this article (doi:10.1186/1471-2164-14-838) contains supplementary material, which is available to authorized users.

## Background

Acetic acid is a by-product of *Saccharomyces cerevisiae* fermentation, and may also result from the metabolism of lactic and acetic acid bacteria. In wines, acetic acid above certain concentrations may affect the course of fermentation, leading to sluggish or arrested fermentations [1–3]. In bioethanol production from lignocellulosic acid hydrolysates, it may also be associated with inhibition of the alcoholic fermentation process, limiting productivity [4]. Due to its toxic effects, acetic acid is also employed as a food preservative, though resistance is often found, causing spoilage of preserved food materials [5]. Mechanistically, it is known that, under glucose repression conditions, acetic acid enters the cells by diffusion and at an acidic pH leads to intracellular acidification, anion accumulation and inhibition of cellular metabolic activity, namely fermentation and growth [2, 6]. Acetic acid can also affect cell viability and lead to a programmed cell death (PCD) process with features similar to mammalian apoptosis, such as chromatin condensation along the nuclear envelope, DNA fragmentation, ROS accumulation, hyperpolarization followed by depolarization of the mitochondrial membrane, exposure of phosphatidylserine on the outer leaflet of the cytoplasmic membrane and release of cytochrome *c* (cyt *c*) from mitochondria [7, 8]. This PCD process can proceed via pathways dependent or independent of the yeast metacaspase Yac1p [9–11]. The finding that acetic acid may induce cell death through a process under genetic control opened the door to novel strategies to manipulate this response. Indeed, elucidating the mechanisms of cell death and of their regulatory pathways has now emerged as a new basis for future breeding strategies aimed at cell survival, of interest for biotechnology. The discovery that acetate triggers apoptotic cell death in cancer cells [12, 13] also reinforced the importance of elucidating the mechanisms underlying this process for the biomedical field.

In the context of biotechnology, there has been a mounting effort to elucidate mechanisms of stress resistance in yeast in order to obtain strains with improved performance. However, most studies on stress responses have been focused on the ability of yeast to divide and grow in the presence of toxic agents. In the case of acetic acid, it has been shown that a large number of genes are involved in the response to acetic acid-induced growth inhibition in *S. cerevisiae*, and that tolerance to growth in the presence of acetic acid depends on different regulatory pathways, involving for instance the Hog1p MAPK and the transcription factor Haa1p [14–17]. Several proteins have also been linked to acetic acid-induced PCD in yeast, such as the genes coding for the ortholog of mammalian voltage dependent anion channel (VDAC), Por1p, and the ATPase subunit, Atp2p, with an anti-apoptotic role, and, among others, the yeast metacaspase Yca1p, ADP/ATP transporter (AAC) proteins, the yeast homolog of mammalian Apoptosis-Inducing Factor Aif1p, and of endonuclease G, Nuc1p, with pro-apoptotic roles [11, 18–22]. However, a broad search for genes involved in acetic acid-induced PCD was lacking.

The present work aimed to identify, at a genome-wide scale, genes involved in negative and positive regulation of PCD induced by acetic acid in *S. cerevisiae*. A functional analysis of a yeast knock-out haploid mutant collection sought to uncover mutants with a sensitive or resistant phenotype, thus identifying genes involved in protection or mediation of acetic acid-induced PCD, respectively, was performed. For this purpose, a screening protocol was developed to assess the effect of acetic acid on cell survival. The optimized procedure was then used to screen the whole yeast knockout haploid mutant collection and genes whose deletion resulted in resistant and sensitive phenotypes were clustered according to their biological function and known physical and genetic interactions. The “Mitochondrial function” class had the highest number of genes in both the resistant and sensitive datasets, reflecting the broadly recognized importance of mitochondrial control for yeast apoptosis. We also found that metabolism is a major regulator of cell death, since impairment of major carbohydrate and amino acid metabolic pathways resulted in increased resistance to acetic acid-induced apoptosis. In addition, several other novel putative targets for the control of acetic acid-induced PCD were uncovered.

## Methods

### Strains

The parental strain *Saccharomyces cerevisiae* BY4741 (MATa *his3*Δ1 *leu2*Δ0 *met15*Δ0 *ura3*Δ0) and the respective EUROSCARF collection of derived deletion mutant strains, containing all the non-essential open reading frames replaced by the KanMX cassette, were used (http://www-sequence.stanford.edu/group/yeast_deletion_project/deletions3.html).

### Screening of the mutant collection for cell death susceptibility and resistance

Cells were initially grown in 96-dot arrays on YPDA medium (yeast extract (1%), bacto-peptone (2%), glucose (2%) and agar (2%)) for 48 h. Then, using a 96-pin replicator, strains were transferred into 96-well plates with YPD (yeast extract (1%), bacto-peptone (2%) and glucose (2%)), and grown for an additional 24 hours at 30°C (no agitation) to be used as inocula. Each strain was then diluted 100 fold in YPD medium using a multichannel pipette (Additional file 1: Figure S1). Afterwards, again using a multichannel pipette, 2 μl were transferred to new 96-well plates containing 150 μL of YPD medium adjusted to pH 3.0 with HCl, and with acetic acid at a final concentration of 400 mM. A 2 M stock solution of acetic acid prepared with distilled water and adjusted to pH 3.0 with NaOH was used. At different times of incubation (100, 300 and 350 minutes), a 96-pin replicator was used to transfer a drop (approximately 3 μl transfer volume) from each well into new 96-well plates containing YPD medium without acetic acid, and the plates were incubated at 30°C for 48 hours. Optical density (OD_640 nm_) of the cultures was then read in a microplate reader (SpectraMax Plus, Molecular Devices) and the absence of any increase in OD_640 nm_ was interpreted as indicative of the absence of viable/culturable cells.

Optical density of the 24 h-growth inocula was approximately the same for all the strains, ensuring that the same cell concentration was used in the treatment plates for all strains. Optical density of the dilution plates was also read after 48 h of growth to control for any variation in final OD, and to confirm that all strains grew to the same extent without acetic acid treatment.

### PI staining, chromatin condensation and fragmentation assessment, and detection of phosphatidylserine externalization

Cell death markers were assessed in *Saccharomyces cerevisiae* BY4741, after 350 minutes of exposure to acetic acid in 96-well plates. Plasma membrane integrity was assessed by propidium iodide (PI) staining as previously described [7]. Briefly, cells were collected, washed, suspended in PBS (137 mM NaCl, 2.7 mM KCl, 10 mM Na_2_HPO_4_ and 1.8 mM KH_2_PO_4_) and stained with 2 μg/ml of PI (Sigma-Aldrich) at room temperature for 10 min, in the dark. Phosphatidylserine (PS) exposure was detected by FITC Annexin-V (BD Biosciences) staining as described previously [23]. Briefly, after cell harvesting, the cell wall was digested with 3% (v/v) glusulase (NEE-154 Glusulase; Perkinelmer) and 7 U/ml lyticase (Sigma-Aldrich) for 80 minutes at 28°C. Subsequently, cells were stained with Annexin-V and PI for 20 minutes, in the dark. To assess plasma membrane integrity and PS externalization, the fluorescence was measured in an Epics® XL™ (Beckman Coulter®) flow cytometer, equipped with an argon ion laser emitting a 488 nm beam at 15 mW. Twenty thousand cells were analyzed per sample and experiments were reproduced independently at least two times. Cells with red [FL-4 channel (488/675 nm)] or green [FL-1 channel (488/525 nm)] fluorescence were considered to have lost plasma membrane integrity or to expose PS on the outer leaflet of the plasma membrane, respectively. To assess chromatin condensation, cells were fixed with ethanol, stained with DAPI (4 μg/ml) for 10 minutes at room temperature, in the dark, and observed by fluorescence microscopy [23]. Cells were visualized in a Leica Microsystems DM-5000B epifluorescence microscope coupled to a Leica DCF350FX digital camera, and at least 200 cells per experiment were counted.

### Cell viability – CFU assays

Cells were grown overnight in YPD medium in an orbital shaker, at 30°C, 200 rpm to OD_640nm_ ≈ 0.5. The strains were then harvested and suspended (10^7^ cells/ml) in the treatment medium consisting of YPD at pH 3.0 (set with HCl) containing 100 mM acetic acid, and incubated in an orbital shaker, at 30°C, 200 rpm. After 100 minutes of treatment, culture samples were taken, diluted, spread on YPDA plates and incubated at 30°C. Cell viability was determined by counting colony-forming units (CFU) that grew after 2 days.

## Results and discussion

### Optimization of the screening protocol to identify genes involved in acetic acid-induced PCD

**Figure 1:**
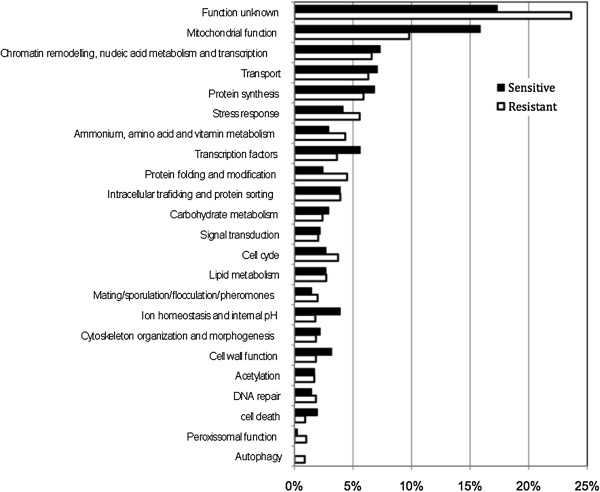
**Functional categories of genes whose deletion renders cells more resistant or more sensitive to acetic acid-induced programmed cell death based on GO and MIPS indexes of biological functions.** The number of genes in each category relative to the total number of genes in the respective dataset, expressed in percentage, is presented.

**Figure 2:**
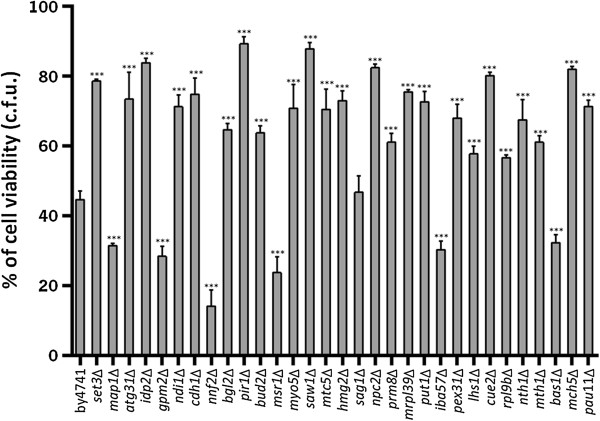
**Cell viability (c.f.u.) of** ***Saccharomyces cerevisiae*** **BY4741 and of 31 isogenic mutant strains tested individually.** Cells were exposed to 100 mM acetic acid in YPD medium at pH 3.0, for 100 min. The *sag1*Δ strain was the only strain whose viability was not statistically different from the wild-type strain. The strains *map1*Δ, *gpm2*Δ, *nnf2*Δ, *msr1*Δ, *iba57*Δ and *bas1*Δ have lower cell viability, while the remaining strains have a higher survival than the wild-type strain. All the results are in accordance with those of the screening. Values represent means and standard deviations of at least 3 independent experiments. Statistical analysis was performed using a one-way ANOVA test. The difference between the wild-type and deletion strains, statistically significant: ***P < 0.0001.

In order to identify genes potentially involved in the positive and negative regulation of acetic acid-induced PCD, we optimized a protocol to screen the EUROSCARF haploid knockout strain collection for yeast mutants with higher resistance or sensitivity to cell death induced by acetic acid than the wild-type strain (BY4741) (Additional file 1: Figure S1). Strains grown in 96-well plates were exposed to 400 mM acetic acid in YPD medium at pH 3.0 for up to 350 min. Mutants that were not viable at a specific time point where the control strain remained viable (100 min) were considered sensitive. In contrast, mutants that remained viable at both time points where the control strain was no longer viable (300 and 350 min) were considered resistant. Under the conditions optimized for the screen, the selected acetic acid concentration (400 mM) was quite high in comparison with other studies characterizing acetic acid-induced apoptosis [7, 18]. This resistance might be related with the high sensitivity of our assay in detecting even very few culturable cells in the treatment medium, as well as with the low oxygen availability, due to lack of agitation of the plates. Since acetic acid can induce both apoptosis and necrosis, depending on the concentration [7], we addressed whether the high acetic acid concentration used in the optimized assay could be inducing necrosis. For this purpose, wild-type cells exposed to acetic acid in 96 well plates mimicking screening conditions (400 mM, for 350 min) were stained with PI and analysed by flow cytometry. Although no viable cells (assessed by their lack of proliferative capacity in YDP liquid medium) were present under these treatment conditions, we observed only a very small number of PI-positive cells (approximately 9%), showing that plasma membrane integrity was still preserved. Cells were also stained with DAPI to assess chromatin condensation and with FITC- Annexin V to assess phosphatidylserine externalization. Both apoptotic markers [23] were detected in the cultures, confirming cell death was occurring by an apoptotic process (Additional file 2: Figure S2). As a control for the validation of our protocol, we individually tested the cell survival of over 40 deletion strains after exposure to acetic acid by counting colony-forming units (CFU assays), as previously described [18]. The strains were chosen in order to include genes from all the functional categories represented in our datasets (see below – Figure 1). All the phenotypes assessed in this manner were in agreement with the phenotypes obtained with the screening experimental procedure (Figure 2 and Additional file 2: Figure S2C).

### Genome-wide identification of determinants associated with sensitivity and resistance to acetic acid-induced PCD

The EUROSCARF haploid knockout strain collection includes approximately ~5,100 strains deleted for each of the nonessential open reading frames (ORF) of *S. cerevisiae*, covering 82% of the ~6,200 annotated yeast ORFs. Growth of a small number of these strains was severely impaired and therefore they were excluded from the results. Of 5080 strains analysed, 2159 were more resistant to acetic acid–induced PCD than the wild-type strain, whereas 409 were more sensitive. The genes whose deletion caused a resistant or sensitive phenotype were manually clustered into functional categories, without repetitions, according to the major function attributed by the Gene Ontology Database and the MIPS Functional Catalogue (Figure 1 and Additional file 3: Table S1). Of these, the most represented was the “mitochondrial function” category, in both the resistant and in sensitive strain datasets. These results are consistent with the known central role of mitochondria in acetic acid-induced PCD [8, 18, 21, 22] and evidence that differential modulation of mitochondrial function can result in either resistance or sensitivity to the acid. However, this category seems to have a higher impact in the dataset of sensitive strains, as do other categories that revealed to be important for resistance to acetic acid–induced PCD, such as the “Transcription factors”, “Ion homeostasis and internal pH”, “Cell wall function” and “Cell death” categories. Some of these categories are also important for growth in the presence of acetic acid, as previously reported in a genome-wide analysis in *Saccharomyces cerevisiae*[16]. On the other hand, several categories displayed a higher percentage of strains in the dataset of resistant mutants, namely the “Stress response”, “Ammonium, amino acid and vitamin metabolism”, “Protein folding and modification”, “Cell cycle” and “Peroxissomal function” categories, suggesting that members of these categories may have a predominant pro-death role in response to acetic acid. “Autophagy” stands out as a category represented exclusively in the dataset of resistant strains.

Multiple genes whose deletion had been shown in previous independent studies to confer resistance or sensitivity to acetic acid-induced PCD were found in our screen. Specifically, among the genes with a mitochondrial function, *ATP10, CYC7, AAC1, NDI1,* and *YSP2* were found in the resistant dataset, and *ATP2* and *POR1* in the sensitive dataset [8, 18, 24–26]. Also present in our resistant dataset were the genes coding for histone Hta1p, yeast neutral sphingomyelinase Isc1p, protease Kex1p, yeast metacaspase Yca1p, ribosome-associated protein Stm1p, rapamycin-sensitive kinase Tor1p, and mitochondrial fission protein Mdv1p, all previously shown to increase apoptotic cell death [9, 27–32]. Several genes with an assigned role in cell death were thus obtained in our genome-wide screen, validating the phenotypic approach developed herein.

### Functional categories significantly enriched in the dataset of sensitive and resistant mutant strains

Through the aforementioned analysis, we gained a general understanding of the gene functions affecting acetic acid-induced PCD. However, in that analysis, the most represented categories may not reflect a higher enrichment, due to their differential representation in the whole genome. In order to determine which functional categories were statistically more significant, we performed a different analysis of our datasets of sensitive and resistant strains using FUNSPEC (http://funspec.med.utoronto.ca/). In this analysis, the frequency of each category represented in our two datasets was compared with the frequency of the same category in the whole genome, according to the Gene Ontology database. The categories that were significantly enriched in our datasets of resistant and sensitive strains (p-value below 0.01) were then identified. The Gene Ontology (GO) categories (which are referred to as “terms” in this database) under the two domains designated “Biological Process” and “Cellular Component” are presented in Tables 1 and 2.

**Table 1:** **Categories that were significantly enriched (p-value below 0.01) based on the physiological function of the genes whose elimination increases the susceptibility to acetic acid**

Category	p-value	Number of genes in the dataset	Number of genes in the category
**Biological Process**			
Mitochondrial translation [GO:0032543]	1.83E-09	23	88
Translation [GO:0006412]	4.51E-06	41	318
ATP hydrolysis coupled proton transport [GO:0015991]	3.76E-05	7	17
Regulation of SNARE complex assembly [GO:0035542]	1.97E-04	4	6
Ion transport [GO:0006811]	2.75E-04	17	107
Protein N-linked glycosylation [GO:0006487]	7.11E-04	8	33
Proton transport [GO:0015992]	7.28E-04	9	41
Protein lipoylation [GO:0009249]	9.01E-04	3	4
Golgi to endosome transport [GO:0006895]	1.12E-03	5	14
Protein complex assembly [GO:0.001261]	1.26E-03	7	28
Proteolysis [GO:0006508]	1.79E-03	12	74
Mitochondrial translational initiation [GO:0070124]	2.15E-03	3	5
Positive regulation of gluconeogenesis [GO:0045722]	2.15E-03	3	5
Group I intron splicing [GO:0000372]	2.26E-03	4	10
Deoxyribonucleotide biosynthetic process [GO:0009263]	4.10E-03	3	6
Vacuolar acidification [GO:0007035]	4.33E-03	6	26
Positive regulation of mitochondrial translation [GO:0070131]	4.82E-03	4	12
tRNA aminoacylation for protein translation [GO:0006418]	4.92E-03	7	35
Regulation of vacuole fusion. non-autophagic [GO:0032889]	6.62E-03	4	13
Positive regulation of transcription from RNA polymerase II promoter [GO:0045944]	8.37E-03	13	100
Mitochondrial respiratory chain complex IV assembly [GO:0033617]	8.83E-03	4	14
**Cellular Component**			
Mitochondrion [GO:0005739]	2.42E-11	118	1072
Mitochondrial large ribosomal subunit [GO:0005762]	1.79E-08	15	43
Mitochondrial ribosome [GO:0005761]	3.90E-06	6	9
alpha-1.6-mannosyltransferase complex [GO:0000136]	5.07E-06	5	6
CORVET complex [GO:0033263]	5.07E-06	5	6
Mitochondrial inner membrane [GO:0005743]	1.31E-04	27	204
Ribosome [GO:0005840]	1.64E-04	36	310
HOPS complex [GO:0030897]	1.97E-04	4	6
Proton-transporting two-sector ATPase complex, catalytic domain [GO:0033178]	4.37E-04	4	7
Ribonucleoside-diphosphate reductase complex [GO:0005971]	9.01E-04	3	4
Mitochondrial matrix [GO:0005759]	1.23E-03	16	111
Large ribosomal subunit [GO:0015934]	2.20E-03	5	16
Ribonucleoprotein complex [GO:0030529]	2.38E-03	32	307
Transcriptional repressor complex [GO:0017053]	3.83E-03	2	2
Mitochondrial proton-transporting ATP synthase, catalytic core [GO:0005754]	3.83E-03	2	2
SPOTS complex (serine palmitoyltransferase) [GO:0035339]	4.10E-03	3	6
Extrinsic to mitochondrial inner membrane [GO:0031314]	5.01E-03	5	19

**Table 2:** **Categories that were significantly enriched (p-value below 0.01) based on the physiological function of the genes whose elimination increases the resistance to acetic acid**

Category	p-value	Number of genes in the dataset	Number of genes in the category
**Biological Process**			
Amino acid transport [GO:0006865]	2.66E-04	25	42
Metabolic process [GO:0008152]	2.87E-04	171	425
Sporulation resulting in formation of a cellular spore [GO:0030435]	4.50E-04	50	103
Oxidation-reduction process [GO:0055114]	5.15E-04	114	272
Cellular response to oxidative stress [GO:0034599]	1.36E-03	34	67
Regulation of cell size [GO:0008361]	1.70E-03	18	30
Meiosis [GO:0007126]	1.73E-03	60	134
Cellular amino acid biosynthetic process [GO:0008652]	1.82E-03	46	98
ATP catabolic process [GO:0006200]	2.59E-03	22	40
Peptidyl-tyrosine dephosphorylation [GO:0035335]	3.14E-03	11	16
Protein phosphorylation [GO:0006468]	4.22E-03	58	133
Filamentous growth [GO:0030447]	6.30E-03	11	17
Amino acid transmembrane transport [GO:0003333]	7.84E-03	14	24
Lipid metabolic process [GO:0006629]	8.47E-03	28	58
**Cellular Component**			
Cytoplasm [GO:0005737]	9.76E-03	733	2026
Set3 complex [GO:0034967]	3.76E-04	7	7
Ribosome [GO:0005840]	3.58E-03	123	310
Vacuole [GO:0005773]	3.89E-03	69	162
Cellular component [GO:0005575]	4.28E-03	260	704
Cytoplasmic membrane-bounded vesicle [GO:0016023]	6,96E-03	7	9

#### Cellular processes involved in negative regulation of acetic acid-induced PCD

##### Mitochondrial function

In the analysis of the genes whose deletion confers sensitivity to acetic acid-induced PCD, and thus with a protective role in this process, “mitochondrion” was the most significantly enriched term, including genes mainly from “mitochondrial ribosomes”, “mitochondrial matrix” and “inner mitochondrial membrane” categories. Grouped under this term were a vast number of genes that encode proteins with a role in respiration, particularly those involved in ubiquinone (coenzyme Q) biosynthesis (*COQ8, COQ9*) and respiratory complex IV (cytochrome *c* oxidase - COX) assembly (*COX11, COX16, COX17, COX18*), and components of respiratory complexes III (ubiquinol-cytochrome *c* reductase complex) (*COR1, CYT1, RIP1*) and V (*ATP1, ATP2, ATP4*). Among the sensitive strains was also the mitochondrial porin, Por1p (known as the yeast voltage-dependent anion channel), essential for respiratory growth, and previously described as a negative regulator of acetic acid-induced apoptosis [18]. It has been demonstrated that COX activity is reduced, the COX2 subunit is degraded and the levels of cytochromes *a + a*_*3*_ are decreased when cells are exposed to acetic acid, which is accompanied by an increase in ROS production, a known mediator of apoptosis triggered by acetic acid in *S. cerevisiae* cells [8]. Although a direct relation between ROS accumulation and loss of cell viability induced by acetic acid is not always observed [25], our results suggest that deletion of the genes identified in our screen might increase ROS accumulation in the presence of acetic acid, and lead to the faster death of the mutant strains.

It was also recently reported that components of the respiratory chain are required to confer protection against acetic acid-induced growth inhibition [16]. Our results further reinforce the prominent role of some mitochondrial functions for yeast tolerance to acetic acid-induced apoptotic PCD.

##### Transcription of glucose-repressed genes

The term “positive regulation of gluconeogenesis” (e.g., *CAT8*, *GCR1*, *TDH1*) also appeared significantly enriched in the dataset of sensitive mutant strains. In agreement, deletion of *SNF1* or *SNF4*, required for transcription of glucose-repressed genes, also results in a sensitive phenotype. This highlights the importance of metabolism regulation, namely repression by glucose, in PCD induction. Since mitochondrial biogenesis and function are under glucose catabolic repression, these results are also in agreement with a protective role of mitochondria in acetic acid-induced PCD.

##### Protein synthesis

A large number of genes whose deletion confers sensitivity to acetic acid encode proteins involved in translation, in the cytosol but mostly in mitochondria, showing the relevance of maintaining protein synthesis active in both cell compartments during acetic acid stress, and again highlighting mitochondrial respiration (or at least some of its components) as an important process in resistance to acetic acid-induced apoptotic PCD.

##### Protein modifications

Perturbations in protein modification, namely N-linked glycosylation, lipoylation, complex assembly and proteolysis also seem to lead to higher sensitivity to acetic acid-induced PCD. Of note, 5 of the 6 genes encoding the proteins from alpha-1,6-mannosyltransferase complex (*ANP1, MNN9, MNN10, MNN11,* and *VAN1*) were present in the dataset of sensitive mutants. This complex is responsible for the addition of mannan to N-linked glycans on proteins at the cis Golgi membrane and as such is involved in the formation of the cell wall and periplasmic space proteins. Defects in the first step of N-glycosylation in the endoplasmic reticulum had previously been shown to induce apoptosis in human cell lines [33] and in yeast [34]. Our results show that further maturation of the N-linked core-oligosaccharide is needed for protection against acetic acid-induced PCD.

##### Vesicular traffic from the Golgi to the endosome and the vacuole

The appearance of the terms “Golgi to endosome transport”, “regulation of vacuole fusion, non-autophagic“, “CORVET complex”, “HOPS complex”, and “vacuole acidification” evidences the importance of the dynamics of vesicular traffic from the Golgi to the endosome and the vacuole for the resistance to acetic acid-induced PCD. This result is in agreement with a previous report where endosome transport and vacuolar degradation were identified as required for resistance to diverse environment perturbations [35].

#### Cellular processes involved in positive regulation of acetic acid-induced PCD

##### Amino acid biosynthesis

Of the genes whose deletion caused resistance to acetic acid-induced PCD, and are thus involved in mediation of this process, “Amino acid transport” was the most significantly enriched term, with “Cellular amino acid biosynthetic process” also significantly enriched (Table 2). Grouped under these terms there were genes encoding proteins involved in assimilation of ammonia, metabolism of urea cycle, creatine and polyamines (e.g. *ALD3, SPE1, SPE3*), metabolism of glutamate (*GDH1* and *GDH2*), metabolism of amino acids of the aspartate family, threonine (e.g. *HOM3, THR1*), arginine (e.g. *ARG1-3, ARG80-82, CPA1*) and methionine (e.g. *MET1, MET2, SAM4*), metabolism of amino acids of the pyruvate family, alanine (e.g. *AGX1)*, isoleucine (e.g. *BAT2)*, leucine (e,g, *LEU1, LEU4*, *LEU9*) and valine (e.g. *LPD1*), metabolism of tryptophan (e.g. *BNA2, BNA3, BNA4)*, histidine *(e.g. HIS2-4,* HIS6, *HIS7)*, glycine (e.g. *AGX1, GCV1, LPD1, SHM2)*, serine (e.g. *SER2, SER3*, *SER33)*, cysteine (e.g. *STR2)* and phenylalanine (e.g. *AAD3-4, ARO1, ARO8-10).* These results show that tolerance to acetic acid-induced PCD is connected with the incapacity of the cell to promote the biosynthesis of amino acids, contrary to what was observed in a study assessing determinants of growth in the presence of acetic acid [16]. These results further support that abrogation of these biosynthetic pathways, some of which are activated in response to acetic acid [28, 36], is beneficial for cell survival. This had already been suggested by the higher resistance resulting from deletion of *GCN2* and *GCN4*, which encode proteins involved in the activation of amino acid biosynthetic genes [28], and is now unequivocally demonstrated. The economy in energy and resources resulting from blocking amino acid biosynthetic pathways may improve the cellular response to the toxic concentration of acetic acid, and thus explain these results.

##### Carbohydrate metabolism

The “Metabolic process” term was the second most significantly enriched in the resistant strain dataset, showing the importance of metabolic control over cell death regulation. Apart from genes involved in amino acid metabolism, discussed above, this class encompasses a high number of genes coding for proteins involved in C-compound and carbohydrate metabolism, namely in the commitment/specific steps of glycolysis and fermentation (*ADH3, ADH4, ADH5, ARO10, ENO1, GLK1, HXK1, HXK2, LAT1, NDE2, PDA1, PDC5, PDC6, PDX1, PFK2, PGM2, PGM3, PYK2, TDH3,* Additional file 4: Figure S3), pentose-phosphate pathway (*GND2*, *RPE1*, *TAL1*, Additional file 4: Figure S4) and citric acid cycle (*ACO1, GDH1*, *GDH2*, *IDH2, MDH1, KGD2*, *KGD1*, Additional file 4: Figure S5), showing that deletion of genes involved in glucose catabolism (both fermentation and oxidation through the TCA cycle and pentose-phosphate pathway) conferred resistance to acetic acid-induced cell death. Similarly, deficiency in the metabolism of the utilization of C2-compounds (e.g. *ADH2, AGX1, ALD4, ALD5, ICL1, MDH2*) and in lipid degradation (e.g. *TGL2, TGL4, TGL5, PLB1, PLB2*) is also associated with higher resistance. Protection associated with blockage of energy production pathways is consistent with energy dependence of an active cell death process.

##### Oxidative stress

The “Oxidation-reduction process” term comprises several dehydrogenases from the “Metabolism” term discussed above, but also members the “Cellular response to oxidative stress” term (e.g. *AFT2, CCP1, GPX1, GRX3, GRX4, GRX6, SOD2, TRX2*). These results may indicate that a certain level of oxidative stress may be beneficial for cellular resistance to acetic acid-induced PCD. A similar hormesis effect was observed for aging yeast, where accumulation of hydrogen peroxide was found to be beneficial to extend chronological life span [37].

##### Cell growth and differentiation

The enrichment in the terms “Sporulation resulting in formation of a cellular spore”, “Meiosis”, “Filamentous growth”, and “Regulation of cell size” suggests that cell proliferation, differentiation and increase in cell size are associated with higher susceptibility to acetic acid-induced cell death. In agreement with this interpretation, the resistant dataset is also enriched in strains with a “Small size” morphology. On the other hand, it is well known that acetate medium is commonly used to induce sporulation, and therefore the results suggest that activation of this pathway in the presence of glucose may signal cell death. Also in agreement with our results, acetic acid-induced cell death in *Candida albicans* was associated with a morphogenic switch from yeast to hypha [38].

A relation between impairment of meiosis and acetic acid resistance is also observed in the food and beverage spoilage yeast *Zygosaccharomyces bailii* ISA 1307, one of the most acetic acid-resistant species known, which forms mitotic but no meiotic spores, further supporting this association [39].

##### Protein phosphorylation

The highly regulated character of acetic acid-induced cell death is also evidenced by the enrichment in the terms “Peptidyl-tyrosine dephosphorylation” and “Protein phosphorylation”, enclosing a high number of genes coding for protein phosphatases and kinases. Mainly, kinases from MAPK signalling pathways and involved in the regulation of metabolism, cell cycle, budding, cell polarity and filament formation are represented. The results showed that a high number of kinases from MAPK pathways play a very important role in regulation of PCD in response to acetic acid, in contrast with what was found for cell proliferation, where MAPK from only two pathways displayed an altered phenotype [17].

##### Autophagosome formation

Kinases involved in the regulation of autophagy induction were also grouped under the term “protein phosphorylation”. Considering that deletion of a high number of genes involved in pre-autophagosomal structure (PAS) formation originated a resistance phenotype (Additional file 4: Figure S6), it appears that the accumulation of autophagic vesicles might increase cell death. Intracellular acidification induced by acetic acid can inhibit vacuole fusion events [40], and no increase in autophagic flux was observed under acetic acid-inducing cell death conditions [25]. Therefore, the energy consumption of starting a process that is then aborted, the accumulation of autophagic vesicles and/or the sequestration of proteins in these structures appear to have a detrimental effect on cell survival. Also with a link to autophagy, under the “Cellular Components” domain, the “vacuole” stands as an important organelle in acetic acid-induced PCD, with a major emphasis in proteolytic functions and in amino acid and cation (protons, calcium, manganese, copper and iron) transport at the vacuolar membrane.

##### Histone deacetylation

Finally, all the genes coding for the components of the Set3 histone deacetylase complex (*CPR1, HOS2, HOS4, HST1, SET3, SIF2, SNT1*) [41] were present in our dataset of resistant strains, showing the relevance of epigenetic control in acetic acid-induced cell death. The Set3 complex is involved in the meiotic-specific repression of early/middle sporulation genes, signals secretory stress through the Mpk1p/Slt2 cell integrity pathway, and has recently been proposed to alter the dynamic deposition and remodelling of nucleosomes containing H2A.Z [41–43]. Here, we show that this complex is also highly relevant in the regulation of acetic acid-induced PCD. The removal of an acetyl group from histone H2B by another deacetylase, Hos3p, has also been described to mediate apoptosis upon acetic acid and H_2_O_2_ treatment [44]. In agreement with these results, we found that the *hos3*Δ strain was also resistant to acetic acid-induced PDC.

### Additional insights into the regulation of PCD and stress responses

**Figure 3:**
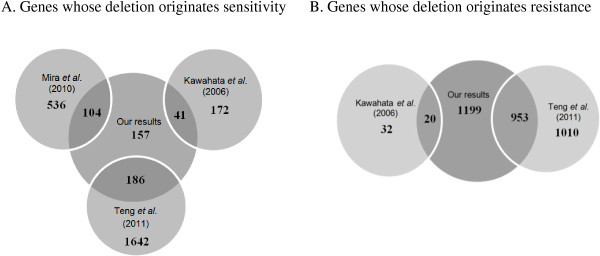
**Schematic representation of the number of common genes found in our own study and in studies of Teng** ***et al.*** [45]**or Mira** ***et al.*** [16]**or Kawahata** ***et al.*** [15]**.** Number of genes with a phenotype in each of these studies and that displayed increased susceptibility **(A)** or resistance **(B)** to acetic acid in our study. Genes with common phenotypes between the studies of Teng *et al.*[45], Mira *et al.*[16] and Kawahata *et al.*[15] are not evidenced in the representation. The numbers in the centre of “Our results” circles represent the number of genes found exclusively in our screen.

A surprisingly high number of knock-out strains (2159) exhibited resistance to cell death in our study. This is, however, in agreement with what was previously observed for cell death induced by heat stress, where approximately 2000 yeast knockout strains were found to be more resistant than the wild-type strain [45]. These results indicate that there are many cellular processes that actively contribute to cell death. From a biotechnological point of view, this information can be very useful for the definition of a “minimal genome” that can significantly increase cell robustness. We therefore compared our results with those of Teng *et al.*[45] to identify the most relevant general functions in cell death mediation in response to both conditions [45]. We found that 60% of the genes from our dataset showed an identical phenotype (either resistance or sensitivity) in response to heat-induced cell death (Figure 3). Analysis of the common genes once again evidences the importance of metabolism in the control of cell death (Tables 3 and 4), and confirms a role for “respiration” in mutant’s sensitivity and for “amino acid metabolism” in mutant’s resistance.

**Table 3:** **Categories that were significantly enriched in the dataset of common genes from our study and the study performed by Teng** ***et al.***[45]**and whose elimination increases the susceptibility to acetic acid and heat**

Category	p-value	Number of genes in the dataset	Number of genes in the category
**Biological Process**			
Mitochondrial translation [GO:0032543]	1.511E-07	12	88
Translation [GO:0006412]	2.893E-07	22	318
Mitochondrial proton-transporting ATP synthase complex assembly [GO:0033615]	8.644 E-04	3	10
Positive regulation of mitochondrial translation [GO:0070131]	1.539 E-03	3	12
Negative regulation of peptidase activity [GO:0010466]	2.353 E-03	2	4
Mitochondrial respiratory chain complex III biogenesis [GO:0097033]	2.353 E-03	2	4
tRNA aminoacylation for protein translation [GO:0006418]	5.078 E-03	4	35
Ion transport [GO:0006811]	5.634 E-03	7	107
Deoxyribonucleotide biosynthetic process [GO:0009263]	5.728 E-03	2	6
Aerobic respiration [GO:0009060]	7.419 E-03	5	61
Chromatin modification [GO:0016568]	7.919 E-03	7	114

In the study of Teng *et al.*[45] a heat ramp stress was applied to the strains of *Saccharomyces cerevisiae* gene knockout collection, after which samples were plated in 96 spots and incubated at 30°C for 18 hours for colony visualization. Microscopic colonies were then assessed by automated counting [45].

**Table 4:** **Categories that were significantly enriched in the dataset of common genes from our study and the study performed by Teng** ***et al.***[45]**and whose elimination increases the resistance to acetic acid and heat**

Category	p-value	Number of genes in the dataset	Number of genes in the category
**Biological Process**			
Oxidation-reduction process [GO:0055114]	1.937E-05	64	272
Metabolic process [GO:0008152]	1.074E-04	88	425
Mannose metabolic process [GO:0006013]	4.085E-04	4	4
Cellular amino acid biosynthetic process [GO:0008652]	9.357E-04	26	98
Cellular response to oxidative stress [GO:0034599]	1.915E-03	19	67
Cytogamy [GO:0000755]	2.229E-03	5	8
Glucose import [GO:0046323]	2.877E-03	3	3
Lipid catabolic process [GO:0016042]	2.888E-03	9	23
Phosphorylation [GO:0016310]	3.096E-03	44	206
Meiosis [GO:0007126]	3.535E-03	31	134
Negative regulation of transcription from RNA polymerase II promoter by pheromones [GO:0046020]	4.429E-03	5	9
Mitochondria-nucleus signaling pathway [GO:0031930]	4.819E-03	4	6
Lipid metabolic process [GO:0006629]	5.704E-03	16	58
Regulation of cell size [GO:0008361]	6.561E-03	10	30
Allantoin catabolic process [GO:0000256]	9.981E-03	4	7

Experimental details used in the Teng *et al.*[45] study are described in the legend of Table 3.

Regarding the dataset of sensitive mutants, the most enriched terms were “mitochondrial translation” and “translation”, evidencing the importance of protein synthesis in coping with both stresses. “N-directed glycosilation” and “ion transport” also seem to have a conserved role in response to both stresses, possibly reflecting common targets. “Negative regulation of peptidase activity” (*PAI3*, *PBI2*) emerges here as a new function in the control of cell death triggered by acetic acid and heat stress, and suggests that proteolytic cleavage by vacuolar proteinases A and B may contribute to cell demise, as described for the human ortholog of proteinase A, cathepsin D. Since Pbi2p is a cytosolic negative regulator of proteinase B, these results suggest that, as shown for proteinase A (Pep4p) in yeast cells treated with hydrogen peroxide or acetic acid [25, 46], proteinase B may also be released from the vacuole under cell death-inducing conditions, and that its activity may also be involved in the cell death cascade. These results are also in agreement with the presence of the *PRB1* gene, coding for proteinase B, in both resistance datasets. In addition, the *PEP4* deficient mutant was found in the dataset of strains resistant to acetic acid-induced PCD. Since this phenotype is in contrast with our previous results obtained in the W303 strain [25], we constructed a new *pep4*Δ mutant strain in the BY4741 background and evaluated acetic acid-induced PCD by C.F.U. counts. The phenotype was confirmed for all the clones tested (not shown). Given the role of Pep4p in mitochondrial degradation [25], the results indicate that Pep4p may play a role in protection or execution of acetic acid-induced PCD, depending on the different mitochondrial mass of the strain background [47].

Analysis of the dataset of resistant mutants uncovered that amino acid metabolism seems to have a more general role in response to cell death, as it is important not only for acetic acid but also for heat-induced cell death. This suggests that heat stress, possibly by affecting the cellular membranes, may also hinder amino acid uptake as described for acetic acid [28]. Like for amino acid metabolism, it is evident from our results that down-regulating glucose metabolism also decreased cell death, evidenced by the appearance of “mannose metabolic process” and “glucose import” terms, which comprise all 3 isoenzymes responsible for initial glucose phosphorylation in glycolysis (*GLK1, HXK1* and *HXK2*). In addition, many of the genes appearing in the “oxidation-reduction process” term code for dehydrogenases involved in carbohydrate, lipid and amino acid metabolic pathways, suggesting that the decrease in NADH/NAD and NADPH/NADP ratios may be involved in signalling cell death. This term shared common genes with the “cellular response to oxidative stress” term, which was also rich in mitochondrial genes, and in genes associated with oxygen and radical detoxification that may lead to a decrease in cellular NADPH levels, and again reduce cell redox potential, namely glutaredoxins (*GRX3*, *GRX4*), thioredoxin II (*TRX2*), thiol-specific peroxiredoxin (*AHP1*) and sulfiredoxin (*SRX1*). Another biological process with a general role in response to both cell death inducers was “phosphorylation”. Under this term, there were many genes coding for kinases from different metabolic pathways and from signalling pathways activated by stress and nutrient availability, but also kinases involved in cell cycle control, consistently with the occurrence of “meiosis” and “regulation of cell size” terms. It has been shown that the mitochondria-nucleus signalling pathway known as retrograde (RTG) response is induced during yeast replicative aging, decreasing cell death and leading to enhanced yeast longevity [48]. However, strains deleted for *RTG1-3* and *MKS1*, major genes controlling this pathway, displayed increased resistance in both screens. Regarding acetic acid-induced PCD, Guaragnella *et al*. [49] have shown that the metabolic state of the cells, namely the level of carbon catabolic repression, can modulate the effect of the RTG pathway in this process, being most evident in raffinose-grown cells, and also affecting resistance of cells grown in 0.5%, but not 2% glucose [49]. Since our results were obtained in 2% glucose, a phenotype resulting from their deletion would not be expected. In addition, since Rtg1-3p and Msk1p are, respectively, positive and negative regulators of the RTG pathway, the fact that deficiency in any of them results in a resistance phenotype suggests that other functions of these proteins, and not only regulation of the RTG pathway, may underlie the phenotypes observed. As an example, Rgt2p is involved in at least two processes in the nucleus, independently of its regulation of Rgt1-Rgt3 transcription factors [50].

As already referred, some of the mutants found as resistant or sensitive to acetic acid-induced PCD in the present screen were also found in previously published screens that aimed to find genes required for growth in the presence of acetic acid [15, 16]. 122 mutants were found in both our study and the study developed by Kawahata *et al.*[15], although only 50% of these mutants showed the same phenotype (16.4% were resistant and 33.6% sensitive to acetic acid) (Figure 3). It is important to refer that Kawahata *et al.*[15] used the BY4742 strain instead of BY4741 and their study was conducted on solid YPD medium at two different acetic acid concentrations and pH values (66.7 mM at pH 4.3 for selecting sensitive strains, and 83.4 mM at pH 4.2 for selecting resistant strains). Regarding the screen reported by Mira *et al*. [16], 279 mutants were found in our study but only 37.3% of these showed a similar phenotype (Figure 3A) [16]. This work was performed with cells in mid-exponential phase and in minimal media with acetic acid concentrations between 70–110 mM (pH 4.5), and identified genes whose deletion resulted in higher sensitivity, but not higher resistance, to growth under these conditions. Regarding the functional categories of the genes identified, there was a good agreement between the three studies, with examples of common categories being “carbohydrate metabolism”, “transcription”, “intracellular trafficking”, “ion transport”, “biogenesis of mitochondria”, “ribosome” and “vacuole”. On the other hand, when comparing the genes identified in our screen and those identified in the previously referred studies, it is noticeable that the largest number of common genes showed a contrasting phenotype. Among these, the categories with the highest number of genes were: “transcription factors”, “transport”, “intracellular trafficking” and “protein sorting” (namely “autophagy” and “transport from and to the Golgi apparatus”), and “ammonium”, “amino acid and vitamin metabolism”, which encompass more genes whose deletion causes resistance in our study and sensitivity in the other two. Taking these results into account, we can propose that these genes are essential for cell growth under sub-lethal acetic acid stress conditions but also appear to have pro-death functions in response to lethal concentrations of this cell death inducer. The fact that different conditions were used in the three screens might also explain the opposing phenotypes observed. Conversely, for the genes giving rise to similar phenotypes, the differences in experimental conditions reinforce the gene’s physiological relevance in mediating either resistance or sensitivity of yeast to acetic acid in general. We found 119 genes whose deletion led to higher sensitivity both to growth in the presence of acetic acid (at least in one screen) and to acetic acid-induced programmed cell death. This list was enriched in genes coding for proteins mainly involved in translation (with many being structural constituents of ribosomes, including mitochondrial ribosomes), ATP hydrolysis-coupled proton transport (components of V-ATPase and mitochondrial ATPase), protein N-linked glycosylation, late endosome to vacuole transport and trehalose biosynthesis (*TPS1*, *TPS2*). 20 strains displayed higher resistance to acetic acid both under growth inhibition or cell death-inducing conditions (Figure 3B). From these, almost half of the strains were deficient in genes coding for mitochondrial and vacuolar proteins, involved in diverse functions such as morphogenesis of the vacuole, intermembrane transfer of phosphatidylglycerol and phosphatidylinositol, assembly of iron-sulfur proteins and stability of the mitochondrial genome. Within the resistant mutants, there were also strains deficient in genes involved in cell cycle and DNA processing, histone deacetylation, carbohydrate and nucleotide metabolism, among others.

## Conclusions

Acetic acid is a normal end product of the alcoholic fermentation by *Saccharomyces cerevisiae*[2]. Over the years, several studies have been developed to better understand how this weak acid affects microorganisms and which resistance mechanisms they develop.

Here, we identified genes involved in the positive and negative regulation of acetic acid-induced PCD in *S. cerevisiae* through a genome-wide analysis. While there was some overlap regarding the functional categories in which deletion strains that showed sensitivity and resistance to acetic acid-induced PCD were included, namely chromatin remodelling, protein synthesis and transcription, specific functional classes such as authophagy, where all mutant strains showed resistance, were also found.

When we compare the functions of genes identified in the present work as relevant for the response to PCD induced by acetic acid and described for PCD induced by heat stress [45] with those identified as required for resistance to growth in the presence of multiple drugs or stress conditions (MDR resistance), we can observe that “translation” seems to be the function with a common determinant role in robustness of the strains in response to both processes (cell growth and cell death). On the other hand, the “amino acid metabolism” function, highly enriched in the two datasets of strains resistant to acetic acid- and heat-induced cell death, included many genes involved in aromatic amino acid biosynthesis, previously identified as required for growth fitness in the presence of multiple drugs or stress conditions [36], showing this process has opposing effects in growth and death processes.

Our results indicate that deficiency in several metabolic pathways, including carbohydrate, lipid, amino acid and vitamin metabolism, lead to a decrease in cell death, suggesting that these processes play a role in PCD that is detrimental for yeast survival. The role of carbohydrate metabolism is particularly interesting, as it may have an equivalent in cell death induced by acetate in tumour cells. Indeed, tumour cells, which display increased glycolysis, over-activation of the pentose phosphate pathway, partially repressed respiration (Warburg effect) and a general increase in biosynthetic metabolic rates [51, 52], are more sensitive to acetate than untransformed cells. Notably, we found that the same alterations confer sensitivity to acetic acid-induced PCD in yeast. This correlation can be explained by the fact that the experimental conditions used in our screen (treatment with acetic acid in the presence of a glucose repressible concentration) mimic the metabolic behaviour of tumour cells (aerobic glycolysis) in yeast. Also relevant for the parallel with tumour cells, we found that mutations in “cell growth and differentiation” genes affecting proliferation lead to higher resistance to acetic acid and that functional mitochondria, usually decreased in tumor cells, have an important protective role in acetic acid-induced PCD. As a whole, the results show that many of the cellular and metabolic features that constitute hallmarks of tumour cells confer sensitivity to acetic acid-induced PCD, potentially explaining why these cells are more susceptible to acetate than untransformed cells and reinforcing the interest of exploiting this process in the field of cancer therapy.

In our study, deficiency in many genes with mitochondrial function resulted in a sensitivity phenotype in response to acetic acid-induced PCD, but there were also many genes coding for mitochondrial proteins whose deletion originated resistance. Considering the dual role of mitochondria in yeast cell life (as an energy and metabolite yielding organelle) and death (as a source of pro-death factors), one might speculate that the genes appearing in the resistant dataset could be those coding for proteins with a dual role; that is, besides their known “day” function (for example, in the respiratory transport chain), they may also be involved in mediation of cell death under unfavourable conditions. This was observed for cytochrome *c*, Aif1p [19], Ndi1p [24], Nuc1p [20] and AAC proteins [18]. An interesting observation regarding genes that code for mitochondrial proteins in the dataset of resistant strains is the appearance of a large number of genes with previous unassigned functions, namely many *FMP* genes.

The biological meaning of a programmed cell death process in unicellular organisms has been the subject of much debate. Although the demise of one cell is obviously deleterious for itself, it has been shown that the nutrients released from a dying cell can favour the survival of other cells, suggesting a beneficial role for the population as a whole [53]. By showing an extremely relevant association between the control of the cell proliferation and cell death, our results shed new light into the evolutionary relevance of cell death in yeast, and its role in the control of the transmission of damage to future generations.

In summary, our screen has uncovered numerous genes and functions putatively involved in the positive and negative regulation of acetic acid-induced PCD. They provide strong starting points for future targeted analyses exploring their involvement in the mechanism of PCD induced by acetic acid. Taking into account that acetate induces apoptosis in colorectal carcinoma (CRC) cell lines through pathways similar to those found in yeast, this study paves the way to further explore the mechanisms underlying acetate-induced cell death and design novel strategies using acetate as a prevention/therapeutic agent in CRC. The new genome-wide analysis here performed also provides new putative targets for the control of acetic acid-induced PCD with obvious biotechnological impact. Indeed, it might allow improving the performance of industrial yeast strains during wine and bioethanol production, and to design new strategies for food preservation by inhibiting or activating the PCD process, respectively.

## Electronic supplementary material

Additional file 1: Figure S1: Scheme of the procedure used for the genome-wide phenotypic screen of the EUROSCARF deletion collection. (DOCX 83 KB)

Additional file 2: Figure S2: Cell death markers in *Saccharomyces cerevisiae* BY4741 and individual viability assay for selected deletion mutants. (DOCX 149 KB)

Additional file 3: **Genes whose deletion renders cells more resistant (indicated as +) or more sensitive (indicated as -) to acetic acid-induced programmed cell death, organized according to their biological functions under the functional categories of GO and MIPS indexes.** (XLSX 156 KB)

Additional file 4: **Schematic representation of metabolites, enzymes and genes from Glycolysis and Fermentation, Pentose-phosphate pathway, TCA cycle and of genes involved in the Regulation of Autophagy.** Genes whose deletion originated resistance to acetic acid-induced PCD are highlighted. (PDF 315 KB)

## Abbreviations

PCD: Programmed Cell Death
ROS: Reactive Oxygen Species
cyt c: Cytochrome *c*
VDAC: Voltage Dependent Anion Channel
AAC: ADP/ATP transporter
PI: Propidium Iodide
CFU: Colony-Forming Units
ORF: Open Reading Frames GO, Gene Ontology
PAS: Pre-Autophagosomal Structure
CRC: Colorectal Carcinoma.
