# Supplementary material for: Genome-wide identification of genes involved in the positive and negative regulation of acetic acid-induced programmed cell death in Saccharomyces cerevisiae
Source: BMC Genomics. 2013 Nov 28;14(1):838. doi: 10.1186/1471-2164-14-838 (PMC4046756; doi:10.1186/1471-2164-14-838)
Supplement: Supplementary file 1 — Additional file 1: Figure S1: Scheme of the procedure used for the genome-wide phenotypic screen of the EUROSCARF deletion collection. (DOCX 83 KB) [file 12864_2013_5541_MOESM1_ESM.docx]

Multi-channel pipette (2 µl)

Multi-channel pipette (2 µl)

96-pin replica platter


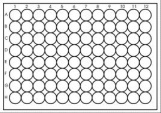

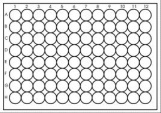

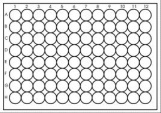


**Treatment plate**

150 µl of YPD medium with acetic acid, pH 3.0

**Dilution Plate**

200 µl of YPD medium

Plate with YPDA media

to growth the cells (48h)

**Inoculum**

200 µl of YPD

medium (24h)

96-pin replica platter


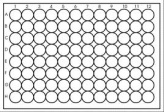

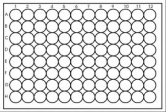

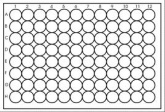


T=350 min.

T=300 min.

T=100 min.

**Additional file 1: Figure S1.** Scheme of the procedure used for the genome-wide phenotypic screening of EUROSCARF mutant collection for the identification of determinants of resistance and sensitivity to acetic acid-induced programmed cell death.
