# Supplementary material for: Genome-wide identification of genes involved in the positive and negative regulation of acetic acid-induced programmed cell death in Saccharomyces cerevisiae
Source: BMC Genomics. 2013 Nov 28;14(1):838. doi: 10.1186/1471-2164-14-838 (PMC4046756; doi:10.1186/1471-2164-14-838)
Supplement: Supplementary file 2 — Additional file 2: Figure S2: Cell death markers in Saccharomyces cerevisiae BY4741 and individual viability assay for selected deletion mutants. (DOCX 149 KB) [file 12864_2013_5541_MOESM2_ESM.docx]

**Additional file 2:
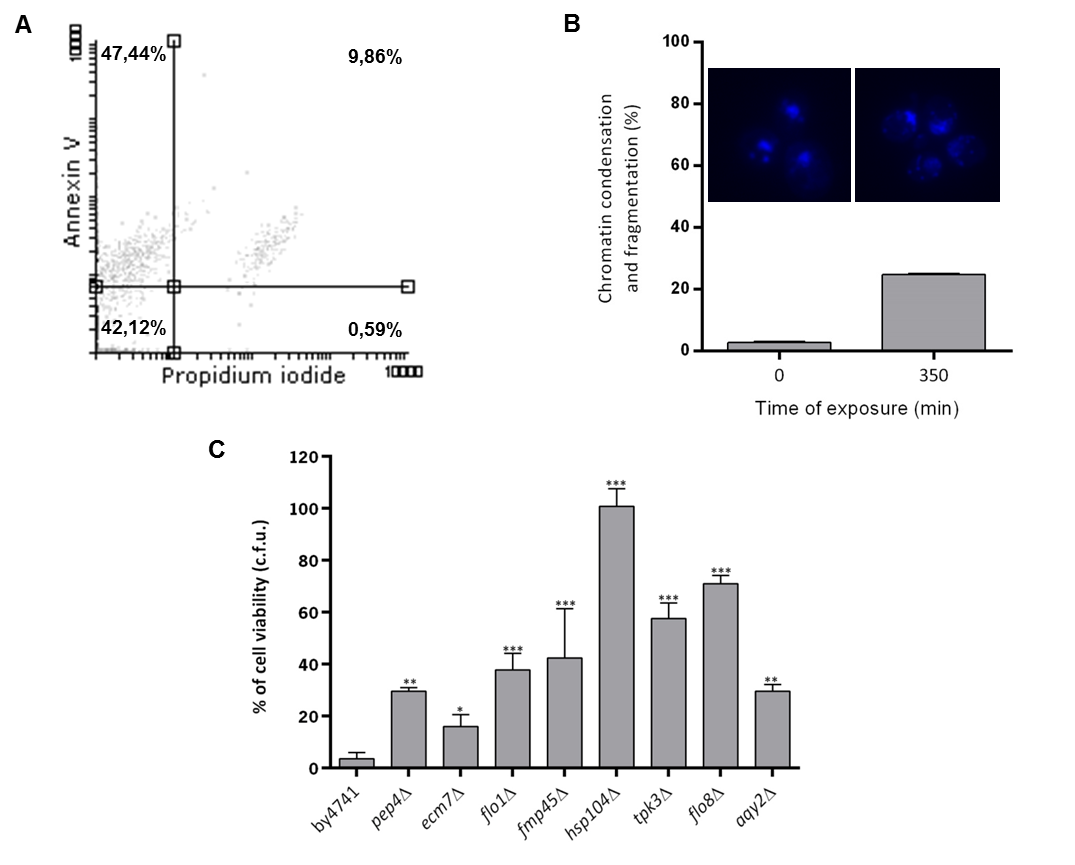
Figure S2.** A-B The appearance of apoptotic markers in the *Saccharomyces cerevisiae* BY4741 strain treated with acetic acid (400 mM) for 350 minutes. A. Flow-cytometric analysis of wild-type cells stained with Annexin V and propidium iodide (PI). Signal exhibited in the different quadrants of the density plot are interpreted as follows: the left bottom population indicate viable cells (Annexin V-/ PI-); left top indicate cells under early-apoptosis (Annexin V+/ PI-); and at right indicate nonviable, necrotic or late apoptotic cells (Annexin V+/ PI+). B. Quantification through fluorescence microscopy of chromatin condensation and fragmentation in wild-type cells stained with DAPI together with photomicrographs indicative of the cells at time zero and after treatment. Results are representatives of two independent experiments. C. Cell viability (c.f.u.) of *Saccharomyces cerevisiae* BY4741 and of 8 isogenic mutant strains tested individually, where the cells were exposed to 120 mM acetic acid in YPD medium at pH 3.0, for 120 min. All the viability results are in accordance with those of the screening. Values represent means and standard deviations of at least 3 independent experiments. Statistical analysis was performed using a one-way ANOVA test. The difference between the wild-type and deletion strains, statistically significant: *P<0.05, **P<0.001 and ***P<0.0001.
