# Supplementary material for: Genome-wide identification of genes involved in the positive and negative regulation of acetic acid-induced programmed cell death in Saccharomyces cerevisiae
Source: BMC Genomics. 2013 Nov 28;14(1):838. doi: 10.1186/1471-2164-14-838 (PMC4046756; doi:10.1186/1471-2164-14-838)
Supplement: Supplementary file 4 — Additional file 4: Schematic representation of metabolites, enzymes and genes from Glycolysis and Fermentation, Pentose-phosphate pathway, TCA cycle and of genes involved in the Regulation of Autophagy. Genes whose deletion originated resistance to acetic acid-induced PCD are highlighted. (PDF 315 KB) [file 12864_2013_5541_MOESM4_ESM.pdf]

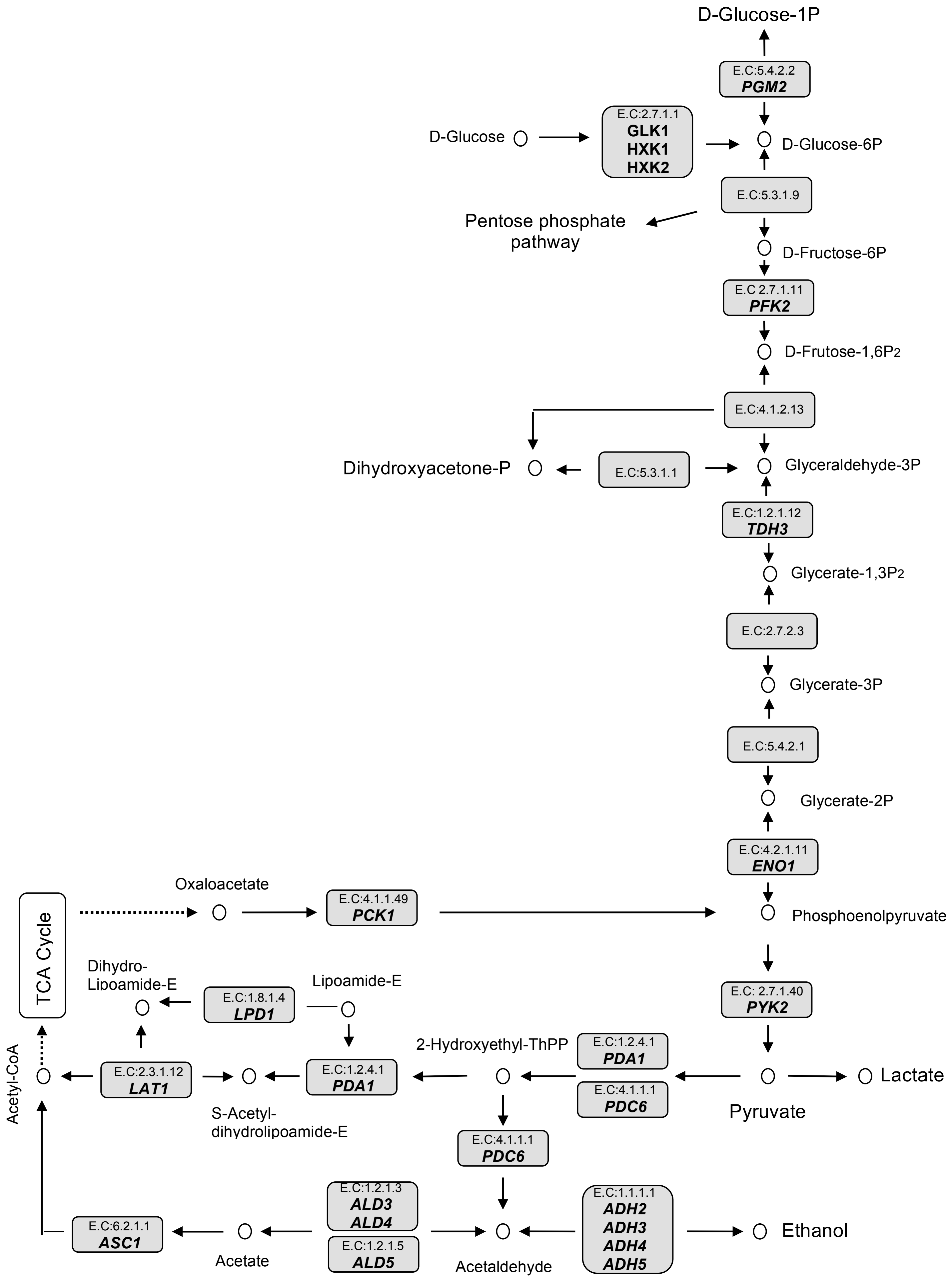

D-Glucose-1P
E.C:5.4.2.2
PGM2
D-Glucose
E.C:2.7.1.1
GLK1
HXK1
HXK2
D-Glucose-6P
E.C:5.3.1.9
Pentose phosphate pathway
D-Fructose-6P
E.C 2.7.1.11
PFK2
D-Frutose-1,6P2
E.C:4.1.2.13
Dihydroxyacetone-P
E.C:5.3.1.1
Glyceraldehyde-3P
E.C:1.2.1.12
TDH3
Glycerate-1,3P2
E.C:2.7.2.3
Glycerate-3P
E.C:5.4.2.1
Glycerate-2P
E.C:4.2.1.11
ENO1
Oxaloacetate
E.C:4.1.1.49
PCK1
Phosphoenolpyruvate
TCA Cycle
Dihydro-Lipoamide-E
E.C:1.8.1.4
LPD1
Lipoamide-E
E.C: 2.7.1.40
PYK2
2-Hydroxyethyl-ThPP
E.C:1.2.4.1
PDA1
Acetyl-CoA
E.C:2.3.1.12
LAT1
E.C:1.2.4.1
PDA1
E.C:4.1.1.1
PDC6
Lactate
Pyruvate
S-Acetyl-dihydrolipoamide-E
E.C:4.1.1.1
PDC6
E.C:1.2.1.3
ALD3
ALD4
E.C:1.2.1.5
ALD5
E.C:1.1.1.1
ADH2
ADH3
ADH4
ADH5
E.C:6.2.1.1
ASC1
Acetate
Acetaldehyde
Ethanol

Figure A3. Schematic representation of metabolites, enzymes and genes from glycolysis/gluconeogenesis and fermentation. Deletion of the genes represented originated a higher resistant to acetic acid-induced PCD.

<u>Legend:</u>

EC:5.4.2.2 - Phosphoglucomutase

EC:2.7.1.1 - Hexokinase

EC:5.3.1.9 - Glucose-6-phosphate isomerase

EC:2.7.1.11 - 6-phosphofructokinase 1

EC:4.1.2.13 - Fructose-bisphosphate aldolase, class I

EC:5.3.1.1 - Triosephosphate isomerase (TIM)

EC:1.2.1.12 - Glyceraldehyde 3-phosphate dehydrogenase

EC:2.7.2.3 - Phosphoglycerate kinase

EC:5.4.2.1 - 2,3-bisphosphoglycerate-dependent phosphoglycerate mutase

EC:4.2.1.11 - Enolase

EC:2.7.1.40 - Pyruvate kinase

EC:4.1.1.49 - Phosphoenolpyruvate carboxykinase (ATP)

EC:1.2.4.1 - Pyruvate dehydrogenase E1 component

EC:1.8.1.4 - Dihydrolipoamide dehydrogenase

EC:2.3.1.12 - Pyruvate dehydrogenase E2 component (dihydrolipoamide acetyltransferase)

EC:4.1.1.1 - Pyruvate decarboxylase

EC:1.1.1.1 - Alcohol dehydrogenase 1/7

EC:1.2.1.3 - Aldehyde dehydrogenase (NAD^+)

EC:1.2.1.5 - Aldehyde dehydrogenase ($NAD(P)^+$)

EC:6.2.1.1 - Acetyl-CoA synthetase

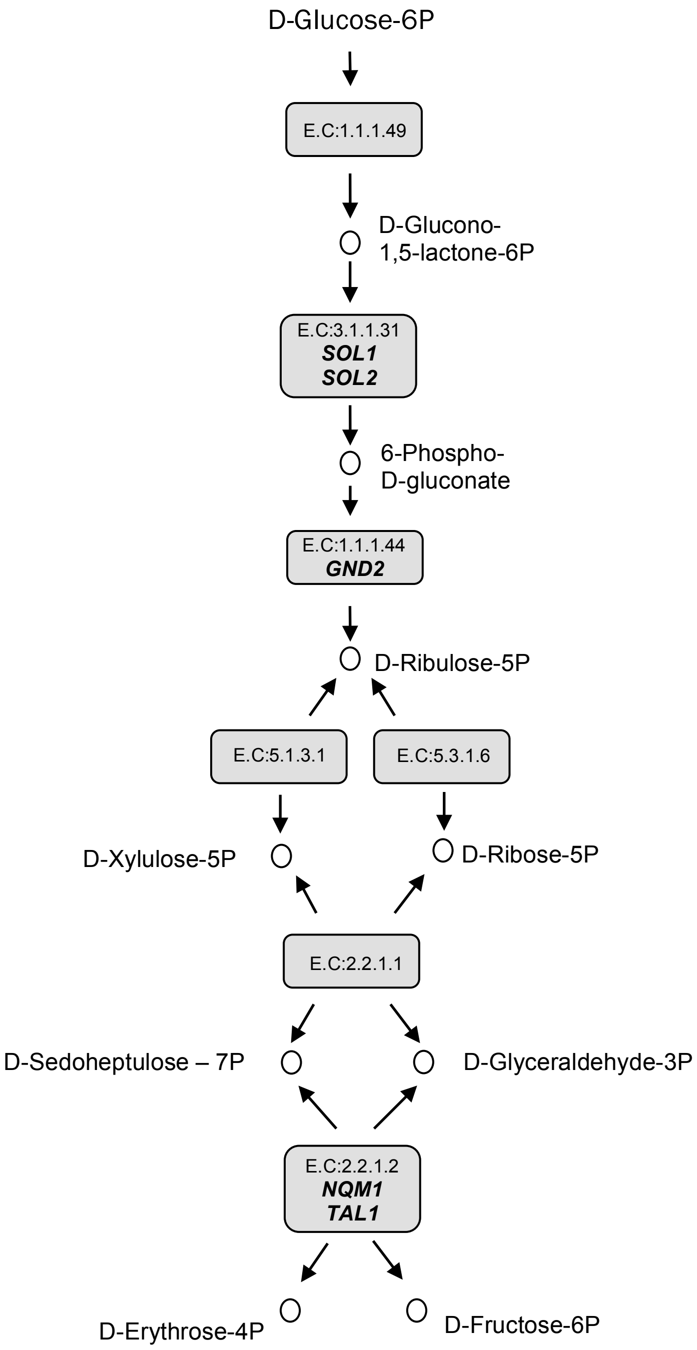

Figure A4. Schematic representation of metabolites, enzymes and genes from pentose-phosphate pathway. Deletion of the genes represented originated a higher resistant to acetic acid-induced PCD.

<u>Legend:</u>

EC:1.1.1.49 - Glucose-6-phosphate 1-dehydrogenase

EC:3.1.1.31 - 6-phosphogluconolactonase

EC:1.1.1.44 - 6-phosphogluconate dehydrogenase

EC:5.1.3.1 - Ribulose-phosphate 3-epimerase

EC:5.3.1.6 - Ribose 5-phosphate isomerase A

EC:2.2.1.2 - Transaldolase

EC:2.2.1.1 - Transketolase

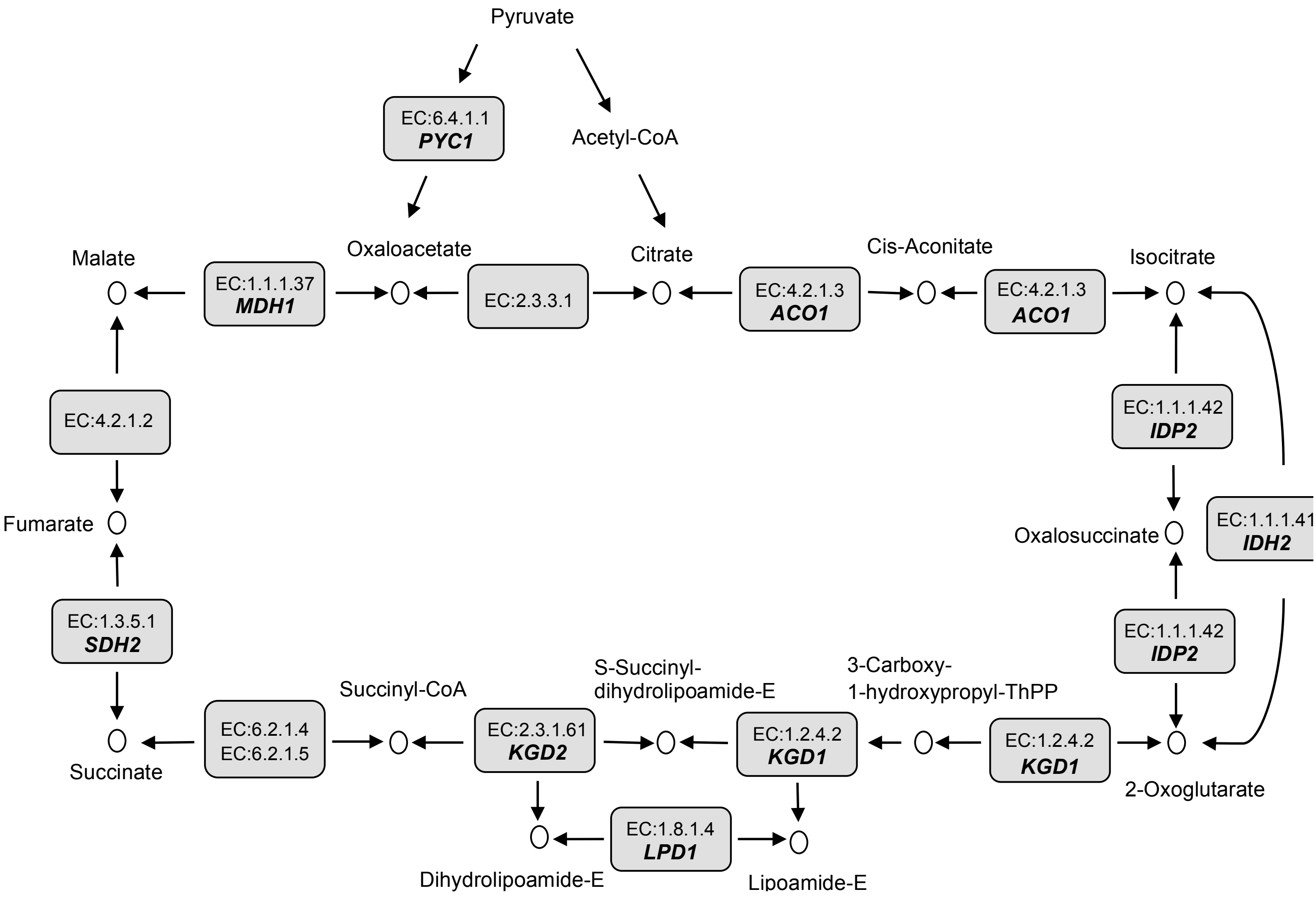

Figure A5. Schematic representation of metabolites, enzymes and genes from tricarboxylic acid cycle (TCA). Deletion of the genes represented originated a higher resistant to acetic acid-induced PCD.

Legend:

EC:6.4.1.1 - Pyruvate carboxylase

EC:1.1.1.37 - Malate dehydrogenase

EC:2.3.3.1 - Citrate synthase

EC:4.2.1.3 - Aconitate hydratase 1 / homoaconitase

EC:1.1.1.42 - Isocitrate dehydrogenase

EC:1.1.1.41 - Isocitrate dehydrogenase (NAD^+)

EC:1.2.4.2 - 2-oxoglutarate dehydrogenase E1 component

EC:1.8.1.4 - Dihydrolipoamide dehydrogenase

EC:2.3.1.61 - 2-oxoglutarate dehydrogenase E2 component (dihydrolipoamide succinyltransferase)

EC:6.2.1.4 - Succinyl-CoA synthetase alpha subunit

EC:6.2.1.5 - Succinyl-CoA synthetase alpha subunit

EC:1.3.5.1 - Succinate dehydrogenase (ubiquinone) flavoprotein subunit

EC:4.2.1.2 - Fumarate hydratase, class I

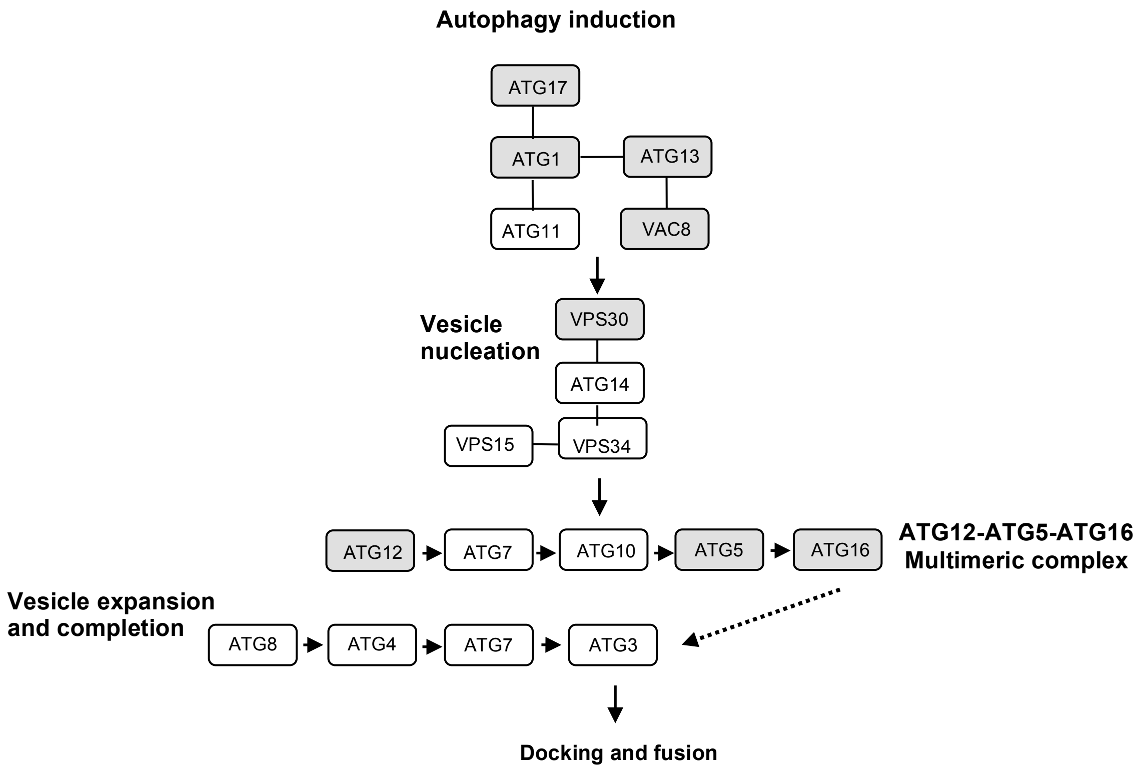

Figure A6. Schematic representation of genes involved in the regulation of autophagy and pre-autophogosomal formation. Deletion of the genes represented in grey originated a higher resistance to acetic acid-induced PCD.
